# Construction of GAN‐RES and Its Application to Small Sample Fusulinid Fossil Recognition

**DOI:** 10.1002/ece3.71845

**Published:** 2025-08-03

**Authors:** Jiahui Xu, Yang Lu, Xu Xu

**Affiliations:** ^1^ Jilin Normal University Siping City Jilin Province China

**Keywords:** deep learning, generative adversarial networks, image recognition, paleofossil recognition, small sample learning

## Abstract

Traditional fossil identification relies on the rich experience and knowledge of paleontologists, and existing intelligent identification methods mainly rely on deep learning to train on a large number of fossil graphic samples to achieve a high degree of precision. In order to solve the above problems and still be able to accurately recognize small samples of rare fossils, we try to use the generative adversarial network (GAN) combined with ResNet50, EfficientNet, and customized CNN architectures, which are applied to the identification of small samples of fossils. First of all, the generator of GAN is fully trained, using it to generate a large number of samples to expand the dataset, enriching the image features extracted by the model, and then through the neural network to analyze the image abstraction computation, and finally, the best fossil identification model is trained through multiple iterations. Using the method of this paper on the same dataset with a data enhancement method for comparison experiments, the experimental results show that the accuracy rate reaches 93% in the case of epochs 100, higher than the other experimental results, and has a significant advantage in the recognition of fossils with scarce samples.

## Graphical abstract

Expanding the dataset using generative adversarial network (GAN) coupled with neural networks to identify small samples of rare fossils.

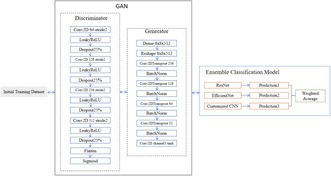

## 1 Introduction

The field of paleontology focuses on the study of fossils. Through the precise identification and analysis of fossils, combined with relevant geological data, personal experience, and professional knowledge, it delves into the evolution of the Earth's environment and the evolution of organisms. This research is of crucial significance for answering fundamental questions regarding the origin and evolution of life on Earth, as well as exploring how biological systems are influenced by the ever‐changing environment and how they, in turn, affect the environment. Traditional certain microfossil image recognition relies on morphological analysis methods, accumulated experience and knowledge reserves of human experts, and vulnerable to subjective factors. Influenced by intraspecific plasticity and phylogenetic homology, there are controversies regarding the validity or diagnostic characteristics of some species, which makes species identification rather challenging. As a result, some researchers fail to “correctly” identify species, and the recognition rate of some genera is below 80% (Hsiang et al. 2019). These issues highlight the requirement for aids or auto ID systems; the systems will follow the morphological thresholds determined by a specific expert, ensuring consistency and accelerating the identification process.

With the development of computer technology, many automatic identification models such as deep learning are combined with paleontological fossil images to extract features from fossil images fed into convolutional neural networks, which are converted into higher level abstract representations through nonlinear models to efficiently deal with image analysis problems. Numerous studies have shown that neural networks have good recognition accuracy for sampled some microfossils and invertebrate groups. This substantiates the applicability of neural networks within this domain, for example, Liu and Song (2020) used four classical deep convolutional neural networks to recognize microfossils and achieved 95% accuracy, the dataset used included public database of 1133 references and 30,815 images from 18 fossil groups (such as sponges, forams, bivalves and corals) and four different kinds of minerals. Wang et al. (2022) designed a Transpose Convolutional Neural Network (TCNN) to further extract different features of fossil images by adding a convolutional layer for up‐sampling to achieve more than 90% recognition accuracy, but when the number of training samples is small, or rather, the number of training samples for each morphological type is small, meaning there are even fewer images for each type, the model's recognition effect is greatly reduced. Marchant et al. (2020) utilized neural networks to classify foraminifera to achieve automatic recognition for this classification with an optimal accuracy of about 90%, the construction of the classifier was based on a large foraminifera dataset of over 34,000 images, Endless Forams (Hsiang et al. 2019).

While all of the above studies have achieved good results, and neural networks still have high recognition accuracy even for less well‐preserved fossils (Bourel et al. 2020), their focus has been on how to improve model accuracy, ignoring the scarcity of the samples themselves. The training of the model relies on the existing taxonomic consensus, and the identification of new taxa or the revision of taxa will still depend on the judgment of professionally trained taxonomists in various fields. On this basis, if the number of some fossils is less than that of extant, more abundant, and more readily available species samples, the model's ability to learn the feature differences between samples will be affected, or overfitting may occur, resulting in poor performance of the model in the testing set or in other applications (Marchant et al. 2020). Furthermore, fossil images are considerably influenced by their formation, burial, and sampling conditions. Meanwhile, since the training data consist of two‐dimensional images (usually thin sections), the image angle or the thin‐section angle can also cause the same species to exhibit vastly different imaging morphologies. All these factors pose a great challenge to the recognition performance of the model (Hou et al. 2023).

In order to study and improve the above problems, the experiments take the foraminiferal fossil order fusulinids as the object of study. The “Fusulinid images 2400—NJU” mentioned in Huang et al. (2023) is the largest fusulinid dataset to date. Hou et al. (2023) conducted studies on this dataset, created grayscale images of the original dataset, and publicly released them. This study uses the images publicly released by this as the experimental dataset. The dataset contains 2400 original images from 16 genera and their grayscale versions, that is, a total of 4800 images. We add a DCGAN (deep convolutional generative adversarial network) structure according to the method of Hou et al. (2023). The generator in DCGAN learns image features and synthesizes photo‐realistic images with identical characteristics for subsequent classification model training. We train the generator by analyzing the input graphic features, so that it constantly fits to approximate the real image and generates a fictitious image expanding the database training model for preprocessing, and then, we use transfer learning to construct the ResNet50 (residual network), EfficientNet and customized CNN classification models to make further improvements to model quality.

The innovation of this method is that, based on the traditional data enhancement, that is, utilizing the original images of different forms, it generates a training model with fictitious images that were not in the dataset and expands the dataset from the source to enhance the capacity of the model to extract image features. The integrated framework of the method is widely applicable to various deep learning classification models as well as fossil images, rather than a specific class.

## 2 Method

### 2.1 Information and Data

The “Fusulinid images 2400—NJU” dataset contains 2400 images from 16 genera, including 295 microscope photographs and 2105 literature scanned PNG images, labeled by their species names and data sources, covering all 6 subfamilies of fusulinids in the taxonomic system. This study utilizes both the original images and their grayscale counterparts (see Table 1). The genus level was used in this study due to the imbalance in the number of image classification samples at this level.

**TABLE 1:** Image classification in fusulinid images 2400—NJU.

Family	Subfamily	Genus	Number
Fusulinidae	Fusulinidae	*Fusulina*	300
Fusulinidae	Fusulinelliinae	*Fusulinella*	300
Fusulinidae	Staffellinae	*Nankinella*	300
Schwangerinidae	Schwangerininae	*Chusenella*	300
Schwangerinidae	Schwangerininae	*Eoparafusulina*	300
Schwangerinidae	Schwangerininae	*Parafusulina*	300
Schwangerinidae	Schwangerininae	*Pseudofusulina*	300
Schwangerinidae	Schwangerininae	*Quasifusulina*	300
Schwangerinidae	Schwangerininae	*Rugosofusulina*	300
Schwangerinidae	Schwangerininae	*Schwangerina*	300
Schwangerinidae	Schwangerininae	*Triticites*	300
Schwangerinidae	Pseudoschwangerininae	*Pseudoschwangerina*	300
Ozawainellidae	Ozawainellidae	*Eostaffella*	300
Schubertellidae	Schubertellinae	*Schubertella*	300
Neoschwagerinidae	Neoschwagerininae	*Neoschwagerina*	300
Verbeekinidae	Misellininae	*Misellina*	300

In the experiments, images of each genus are partitioned into initial training, validation, and test sets at a 7:1.5:1.5 ratio; the training and validation sets are used to determine model parameters, while the test set serves to assess the model's generalization performance.

The training and validation set images are fed into the DCGAN model, and preliminarily, the dataset is expanded using preprocessing methods such as rotation to adequately train the generator and save the resulting indistinguishable images. We evaluated a traditional neural network model, namely ResNet50 (He et al. 2016), EfficientNet (Tan and Le, n.d.), and a customized CNN, which achieved excellent classification scores on ImageNet. In this experiment, the training set images are fed into the DCGAN model, and the generator is trained with a discriminator loss value of around 50%, that is, the generator produces images that are difficult to distinguish from the real ones. In this experiment, the generator training of the DCGAN model is completed when the discriminator loss reaches approximately 50% (indicating the generated images are indistinguishable from real ones). This process employs transfer learning to reduce training costs and preserves the model with the highest recognition accuracy. Subsequently, images produced by the generator are incorporated into model training to obtain final parameters such as average accuracy.

Subsequently, in the classification model, the training set images processed according to Hou et al. (2023) and the generated fictitious images are fed into the model, and in the process, transfer learning is used to reduce the model's burden and save the model with the highest recognition accuracy and to test the model's accuracy on the testing set and other parameters.

### 2.2 Construction of GAN‐RES Network

Generative modeling is an influential tool for unsupervised learning, which is characterized by the ability to learn the data distribution of the input unknown sample data for modeling and then generate samples following the same distribution to expand the dataset. With the rapid development of neural networks, Goodfellow et al. (2014) proposed GAN.

**FIGURE 1:**
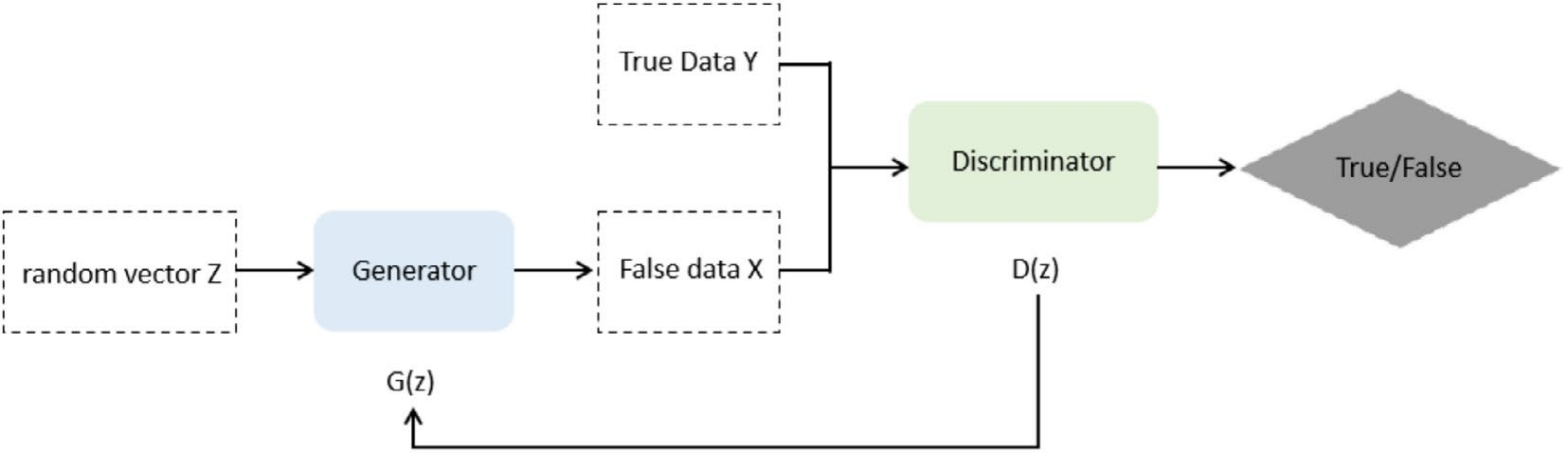
Generate adversarial network structure.

The generative adversarial network consists of two parts, the generator and the discriminator, denoted by G and D, respectively, as shown in Figure 1; input to G is a random vector p_z; and the output of G is a sample G (z) sampled from some distribution p_x. Assume the true data distribution is denoted as p_data. The generator G learns an approximation of this distribution and synthesizes realistic synthetic data to mislead the discriminator D. The input to D comprises two components: authentic samples drawn from p_data and synthetic samples generated by G. They keep training against each other until they reach a relatively balanced state.

**FIGURE 2:**
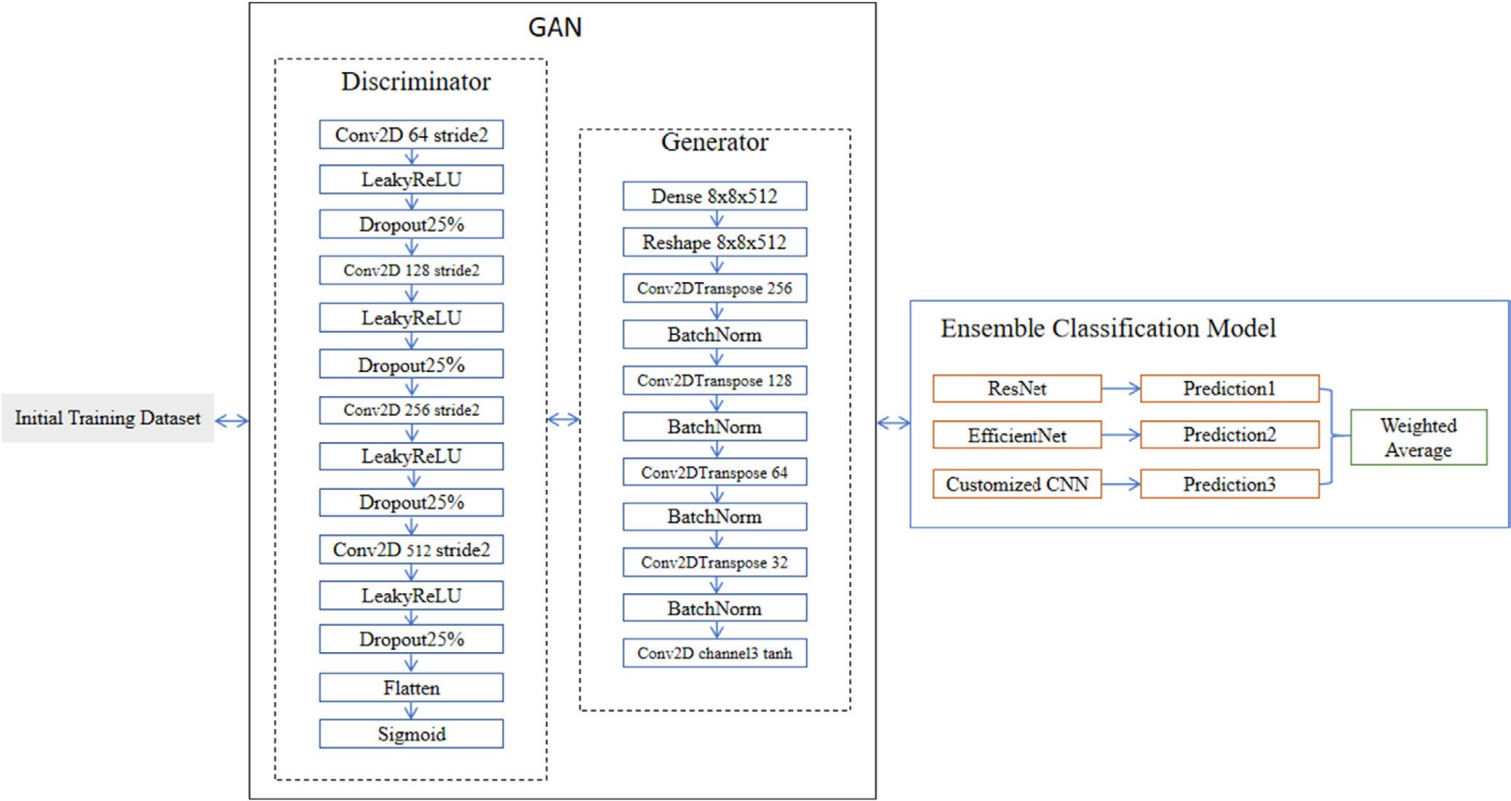
Construction of GAN‐RES and its recognition process.

The current adversarial networks built on the convolutional neural network structure have a generator consisting of a multilayer inverse convolutional network and a discriminator consisting of a multilayer convolutional network. Radford et al. (2016) proposed that the DCGAN is the first model to adopt this structure, where the batch regularization trick was added to assist GAN training, giving it a more stable and good performance. In this fossil recognition process, DCGAN receives the training set image, discriminates its D and generator G for confrontation processing, until the false image generated by G has a recognition loss value of 50% in D, that is, it cannot be recognized, and at this time, the model generating the image with the best image effect is saved. Then, use ResNet50, EfficientNet, and customized CNN to recognize the aggregated true and false images for processing; the overall process is shown in Figure 2.

In EfficientNet, the first 80% of layers remain frozen, while in ResNet5050, the initial 71% of layers are frozen. The custom CNN comprises four convolutional blocks with 3 × 3 kernels, with Adam employed as the optimizer across all architectures. EfficientNet delivers efficient feature extraction, ResNet5050 provides deep feature learning, and the custom CNN captures domain‐specific feature representations. The ensemble system ultimately calculates a weighted average accuracy through a strategic weighting scheme.

### 2.3 Data Preprocessing

The deep learning model has certain standard requirements for the input image data, in order to ensure that the user inputs any size of the image can be recognized and processed by this network. In the model, all the images are adjusted to a uniform size of 128 × 128. In addition, in order to improve the model's generalization performance, we first used traditional data enhancement methods (Perez and Wang 2017) such as rotating and flipping and then randomly adjusted the image brightness and contrast (Liu and Song 2020; He et al. 2016; Simonyan and Zisserman 2015; Szegedy et al. 2015), which are not the main recognizable features of the fossil images (Shorten and Khoshgoftaar 2019). These traditional data augmentation methods can effectively expand the dataset and train the model. Subsequently, the images are normalized to improve the training performance of the model. A normalization process is performed. Finally, a one‐to‐one correspondence is established between the image and one of the labels to obtain the processed dataset for use in the model.

### 2.4 Training Methods

Achieving accurate identification of biological fossils using CNN requires a vast dataset as a foundation, so combining transfer learning training to accelerate model convergence on the basis of DCGAN‐generated image expansion dataset is a suitable approach for small dataset training tasks (Brodzicki et al. 2020; Koeshidayatullah 2020). In transfer learning, rather than training the CNN are randomly initialized and the parameters are initialized as obtaining them from pretraining of recognition tasks with large datasets. Therefore, transfer learning reduces computational cost, improves feature extraction, and accelerates model training convergence.

Specifically, for the DCGAN model in the first stage, the size of all original images is unified to 128 × 128, the number of batches is set to 50, and the learning rate is changed step by step using the StepLR method to make the model fit faster and prevent the overfitting phenomenon caused by the improper learning rate to preserve the best model. The model is then used to generate 16 classes of fitted fossil images, respectively, for subsequent classification model training.

For the classification model in the second stage, loading its pretrained model on ImageNet as well as its parameters and subsequently fine‐tuning the network parameters, that is, first preserving the network structure and parameters of certain layers, then adding fossil images and retraining the network structure of the remaining layers and fine‐tuning the parameters (Oquab et al. 2014; Tan et al. 2018). It greatly reduces the amount of data required for neural network automatic identification and expands the application scenarios for subsequent automatic identification.

### 2.5 Assessment of Indicators

We calculated several metrics, such as precision and recall, to evaluate the performance of each individual experiment on the testing set. The aim of this study is to generate simulated images based on fewer original images to identify fossil genera as accurately as possible, which implies that recall and precision are equally important, so the coordinated mean of the two, F1‐score, is introduced to better assess the performance of the model. The F1‐score is a composite metric, that is, the harmonic mean, which is computed by the formula of 2 × precision × recall/(precision + recall).

## 3 Result

**FIGURE 3:**
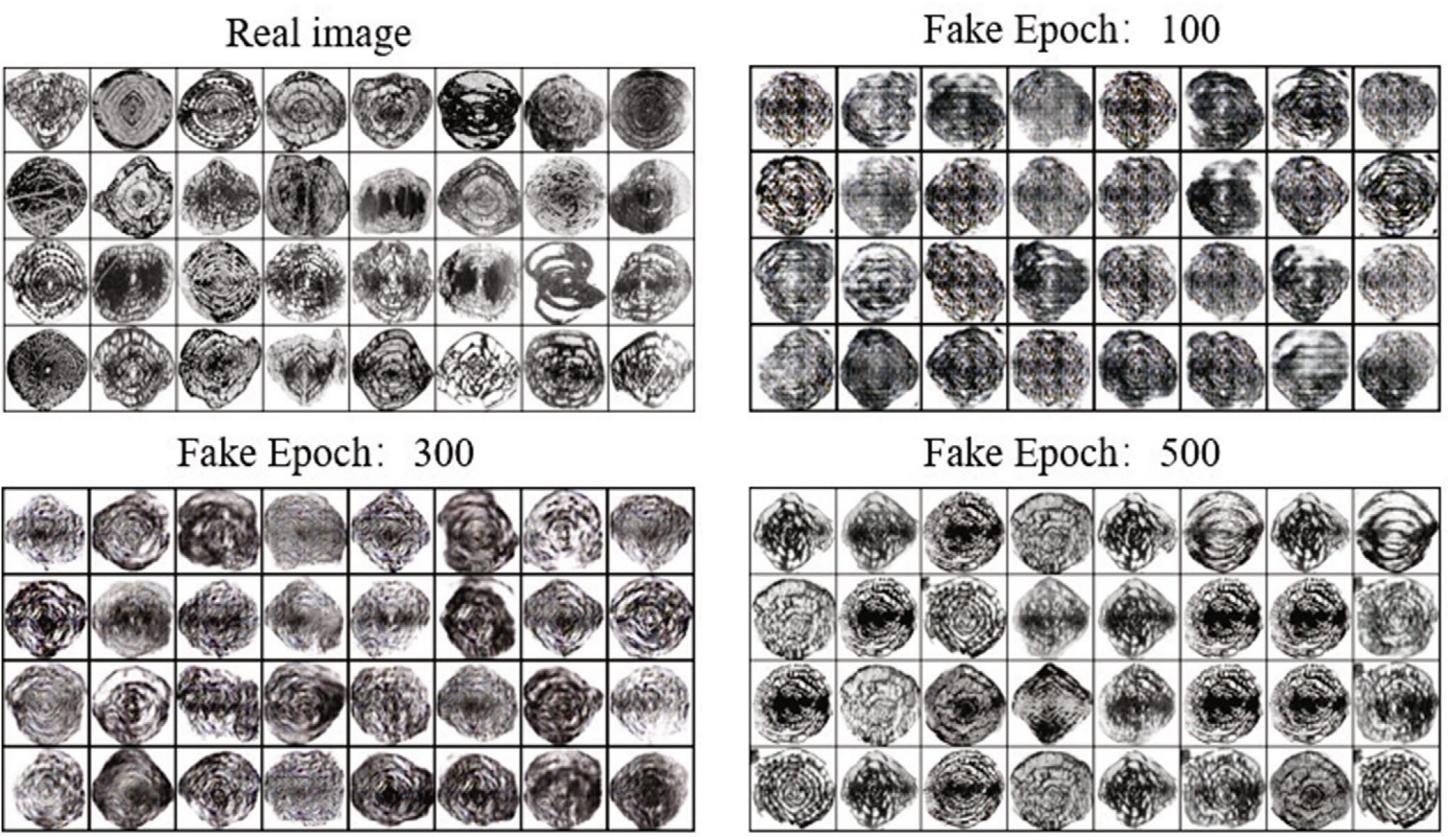
DCGAN: Generated image results with epochs of 100/300/500.

For the first stage of DCGAN, all the original images of fossils are fed into the model, and the best model is saved after 500 rounds of training, which has the optimal values of weights and hyperparameters suitable for the recognition of fossils of those 16 genera, and the random images generated at the 100th, 300th, and 500th rounds are shown in Figure 3, respectively.

**FIGURE 4:**
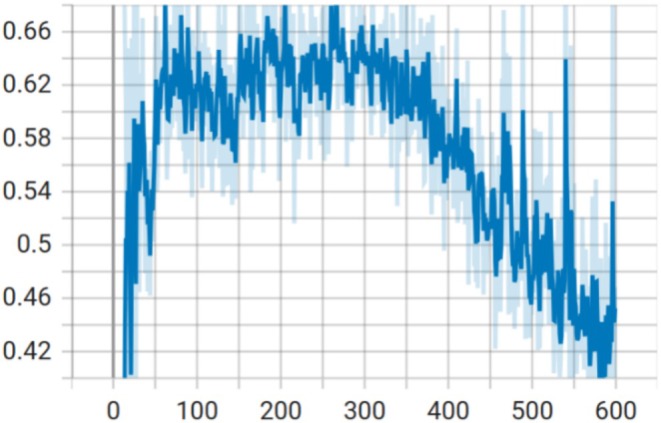
Loss values of discriminators in DCGAN.

When the model is trained for 500 rounds, the discriminator is no longer able to recognize the model between true and false images, that is, the discriminator loss value converges at around 50%, as shown in Figure 4. At this point, the model is saved and the generator of this model is utilized to generate 1000 images for each genus separately, which are added to the subsequent dataset of the classification model in the second stage.

### 3.1 Modeling Performance

The model architecture was tested in the Fusu dataset, achieving an average accuracy of approximately 93% over 100 training epochs with DCGAN‐generated images, as shown in Table 2. However, the data of the images generated by the model are limited. As can be seen from metrics such as precision, there is a risk of overfitting for some genera and species. Future research should focus more on the quality of the generated images. Nevertheless, the overall results prove that this method is still feasible for expanding the dataset.

**TABLE 2:** Various evaluation metrics for GAN‐RES models.

Classes	Precision	Recall	F1‐score
Chusenella	0.92	0.98	0.95
Eoparafusulina	0.92	0.85	0.88
Eostaffella	0.99	0.99	0.99
Fusulina	0.95	0.99	0.97
Fusulinella	0.88	0.91	0.89
Misellina	0.99	0.99	0.99
Nankinella	0.99	0.99	0.99
Neoschwagerina	0.95	0.99	0.97
Parafusulina	0.88	0.87	0.88
Pseudofusulina	0.72	0.80	0.76
Pseudoschwagerina	0.94	0.99	0.96
Quasifusulina	0.94	0.99	0.96
Rugosofusulina	0.85	0.78	0.81
Schubertella	0.99	0.91	0.95
Schwagerina	0.72	0.80	0.76
Triticites	0.85	0.89	0.87
Accuracy	0.93		

## 4 Discuss

### 4.1 Analysis of Appraisal Results

The automatic recognition performance of the trained architecture is comparable to general tasks, suggesting that it is feasible to apply DCGAN‐generated images to AI models in the field of paleontology. The recognition accuracy in genus mode is similar (Romero et al. 2020).

**FIGURE 5:**
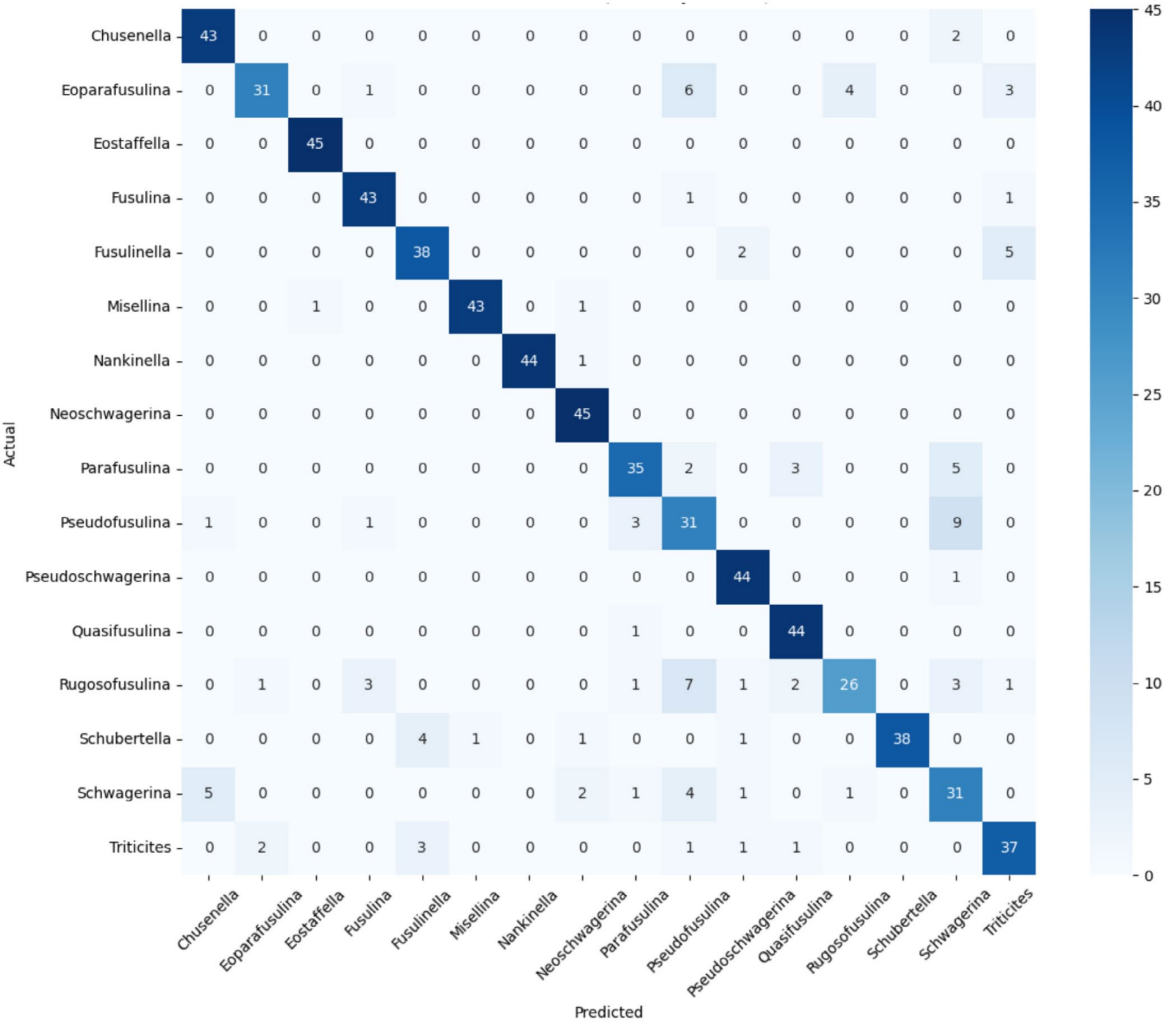
Confusion matrix of the GAN‐RES model for the testing set.

In this experiment, an average accuracy of 93% was achieved among 16 genera. Among them, due to morphological similarities, there is some controversy among experts regarding the classification of eight genera within the family Chwagerinidae of the order Fusulinida. Examples include the genera *Pseudofusulina* and *Schwagerina*, *Schwagerina* and *Chusenella*, which is also reflected in this experimental model. The confusion matrix in Figure 5 shows that the genus that occupies the largest misidentification rate among the *Pseudofusulina* genera is namely *Schwagerina*, and *Chusenella* has the largest number of images misidentified as *Schwagerina*.

**FIGURE 6:**
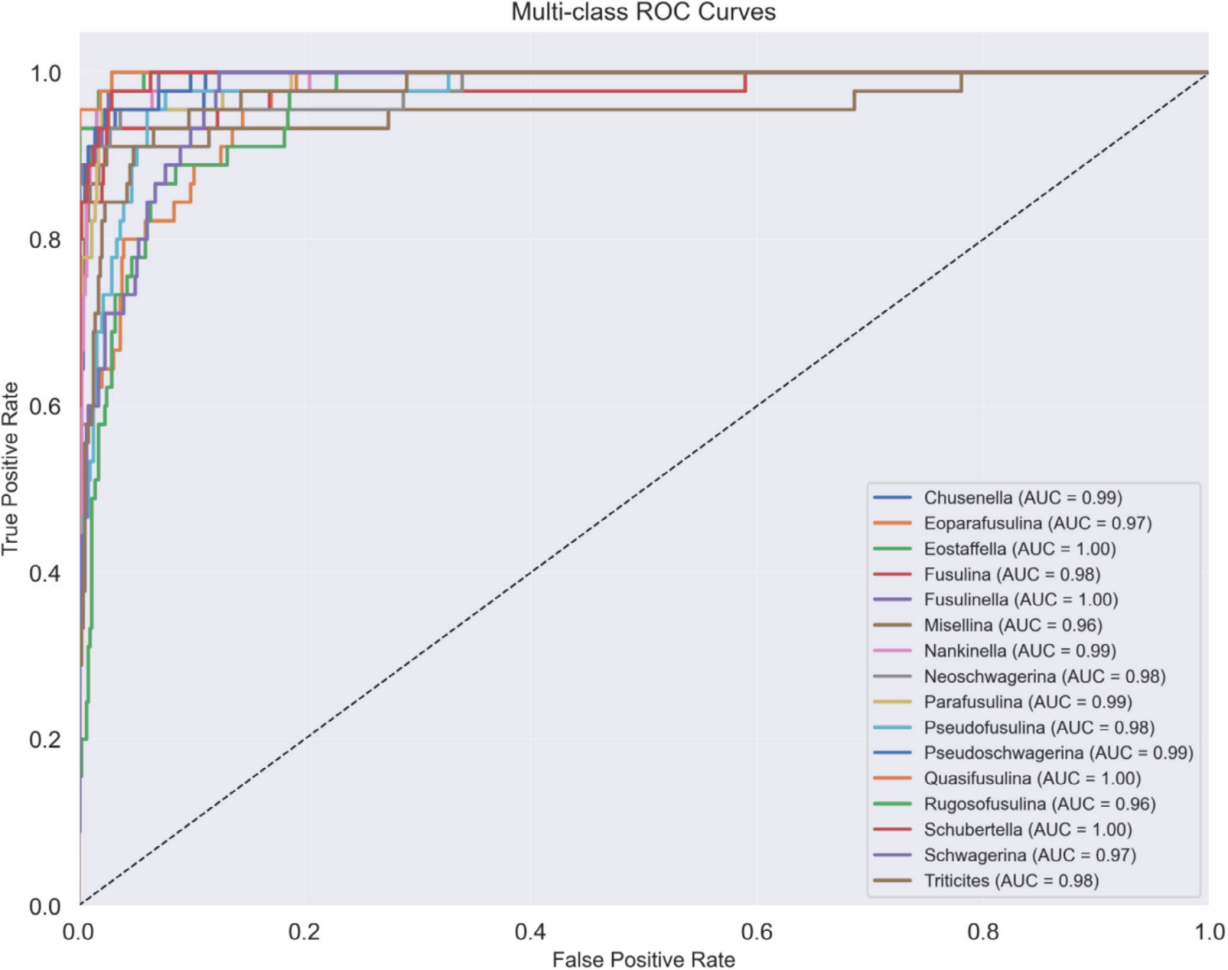
ROC curves of the GAN‐RES random model for the testing set.

In addition, Hou et al. (2023) demonstrated that, by comparing model results with expert identification, the results proved that, except for the poorest consistency between *Pseudofusulina* and *Schwagerina*, there are taxonomic disputes and easy confusion between *Eoparafusulina* and *Triticites* due to their morphological similarities. The degree of confusion for both pairs is around 12%, and the results of these two pairs are reflected in the confusion matrix. The recognition performance for each category is demonstrated in the ROC curves of Figure 6.

### 4.2 Two‐Dimensional Feature Visualization

**FIGURE 7:**
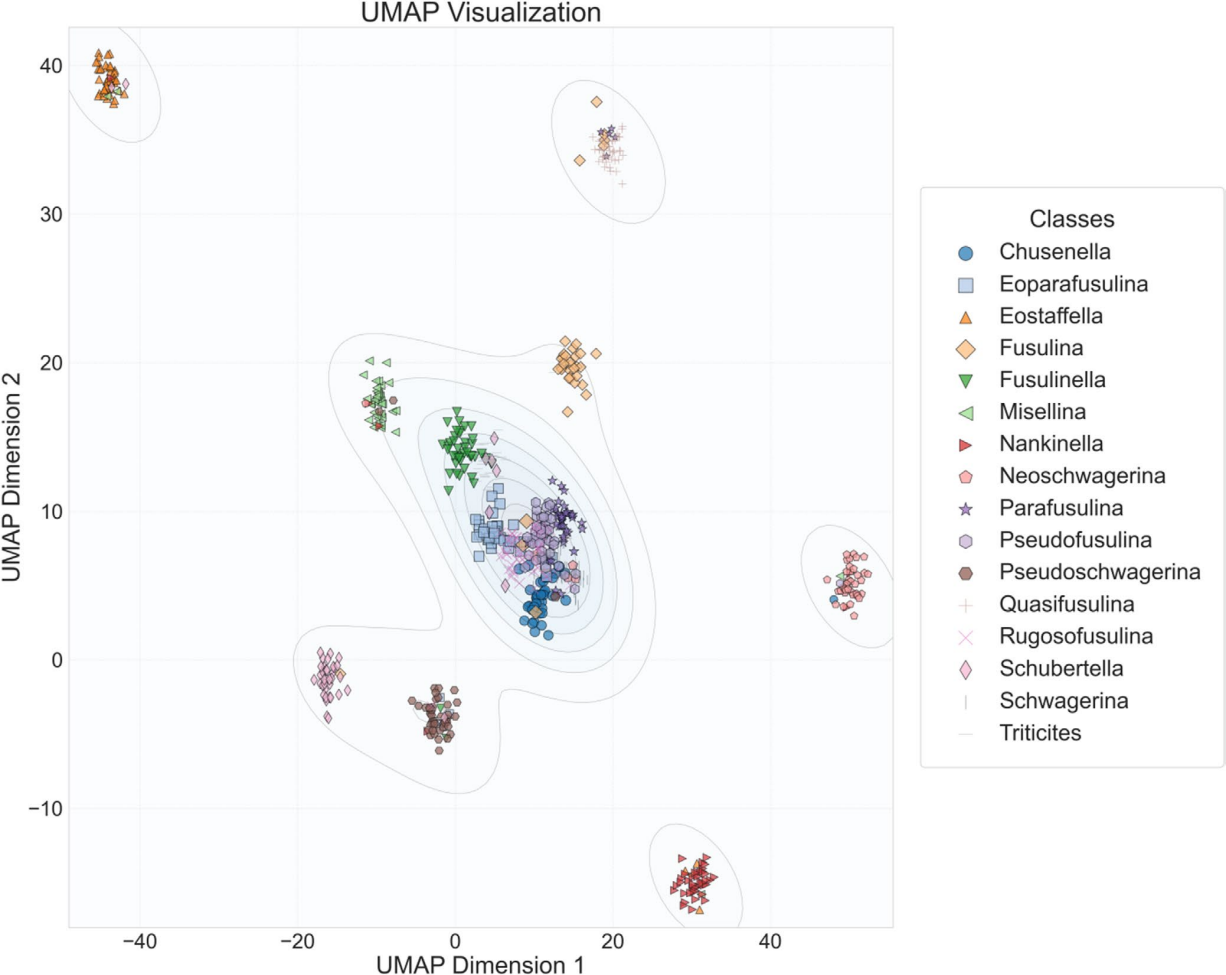
Two‐dimensional feature distribution of genera generated by the GAN‐RES model.

Neural networks have shown excellent results and extremely high efficiency in solving sophisticated vision tasks, but they are at the same time regarded as black boxes that cannot explain their inner workings. For this reason, many methods to explain the working principle of CNNs are developed nowadays, such as UMAP (McInnes et al. 2020), which visualizes images in reduced dimensions and draws images based on the distribution of similarity of image features as shown in Figure 7. In this experiment, UMAP was applied to process high‐level semantic features extracted by the model, thereby more effectively revealing the category distribution patterns learned by the system. The critical hyperparameters were configured as follows: n_neighbors = 15, min_dist = 0.1.

It can be seen that, easily confused fossil genera with similar features such as *Pseudofusulina* and *Schwagerina*, *Schwagerina* and *Chusenella*, and *Pseudofusulina* and *Triticites* are distributed in close proximity to each other, or even partially overlap. The accuracy and validity of this distribution, likewise, depend on whether uniform manifold approximation and projection (UMAP) can accurately analyze all the features presented in each fossil image in Figure 7.

While extraneous background noise may contaminate the test image, the neural network model architecture can fetch representative regions.

**FIGURE 8:**
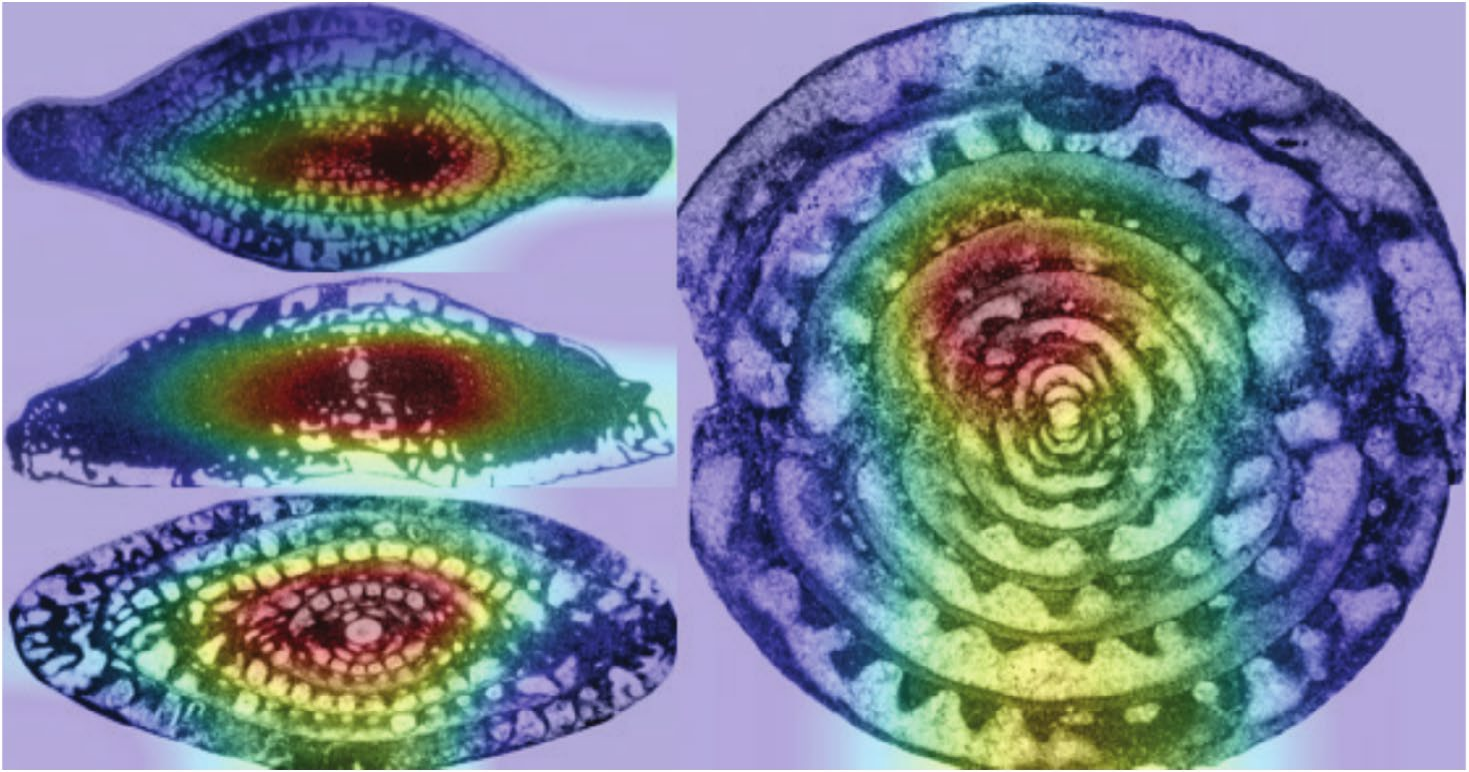
GAN‐RES model for feature extraction region in real images.

Selvaraju et al. (2020) proposed a method called GradCAM to visualize class distinctions and localize image regions associated with classification (Figure 8). Since the training dataset contains two‐dimensional planar features, the model analyzed which regions and features are crucial for the identification of different fossils from a structural perspective.

The model may use some features for recognition that are not used by the expert, and it is possible that this occurs because of the variety of variation between the two groups of taxa, with the expert and the model choosing different views of the features. This experimental model establishes a correspondence between inputs and outputs, and its results show that the results of automatic taxonomic identification are closely related to fossil morphology (Sun et al. 2023).

The fusulinid classification is mainly based on their wall (named “spirotheca”) structure and internal endoskeletons such as folding state of the chamber partitions, development of the calcite double ridges in the central area, or multiple ridges in the chambers. This experimental model establishes a correspondence between inputs and outputs, and its results demonstrate that the outcomes of automated taxonomic identification exhibit a strong correlation with the aforementioned fossil morphological characteristics.

Therefore, neural network models can efficiently extract image features, mitigating the impact of human labeling and reducing its time cost. On the other hand, deep learning can automatically extract fossil features. When dealing with images on a large‐scale dataset, it can obtain more specimen information to make up for the shortcomings of manual identification.

To summarize, GAN‐RES can effectively extract texture features such as curves and boundaries from images, generating anamorphic images to expand the dataset and improve the recognition accuracy of traditional neural networks. While class activation and feature maps give a better understanding of their important feature regions, plainly, how the model handles fossil images.

## 5 Conclusion

Deep learning can extract and analyze the complex features of fossils, reduce the dimensions of images for in‐model processing, and accelerate the experts' fossil classification and identification process. Moreover, this process is more efficient than manual feature selection (Keçeli 2017). In this study, considering the limitations encountered when conducting research on small samples of rare fossils in paleontology, machine‐learning techniques are adopted to assist experts in fossil identification, thus making up for these deficiencies.

The researchers in Hou et al. (2023) previously proposed a multiview integration framework for the dataset used in this paper. They trained multiple models by collecting different views of each fossil specimen for recognizing fossil images. As a result, this approach achieved an average accuracy of about 93% on the model. In this experiment, the GAN‐RES network framework is constructed by DCGAN combined with the data enhancement method of multiple views to generate the anamorphic image expanded dataset.

We finally achieved a recognition accuracy of greater than 93% among 16 genera, which indicates the importance of data volume for neural network training models, and DCGAN can effectively compensate for the lack of data and improve the training accuracy of the model.

With the development of research on paleontological taxa, we can use deep learning models to assist human labor in conducting further quantitative morphological assessments, thereby accelerating the research process (De Baets 2021). This study provides a method to artificially increase the size of training datasets and train some models. These models can reduce the identification time of large foraminifera fossil datasets used in paleoecological and paleoenvironmental studies, enabling senior paleontologists to engage in other more creative research. However, for researchers studying new taxa, we still need to rely on manual assessment of the materials when evaluating their significance (Sun et al. 2023).

## Author Contributions

**Jiahui Xu:** conceptualization (equal), data curation (equal), formal analysis (equal), investigation (equal), methodology (equal), resources (equal), software (equal), visualization (equal), writing – original draft (equal). **Yang Lu:** methodology (equal), resources (equal), supervision (equal), validation (equal), writing – review and editing (equal). **Xu Xu:** funding acquisition (equal), supervision (equal), validation (equal).

## Conflicts of Interest

The authors declare no conflicts of interest.

## Data Availability

All the required data are uploaded as Supporting Information.
